# Supplementary material for: Metastasis of colon cancer requires Dickkopf-2 to generate cancer cells with Paneth cell properties
Source: eLife. 2024 Nov 13;13:RP97279. doi: 10.7554/eLife.97279 (PMC11560131; doi:10.7554/eLife.97279)
Supplement: Supplementary file 1. [file elife-97279-supp1.docx]

**Supplemental Information**

**Table 1. The list of primers used in quantitative real time PCR**

| *Dkk2* | Forward (5’ -3’) | GTACCCGCTGCAATAATGGAATCT |
| --- | --- | --- |
|  | Reverse (5’ -3’) | AACAGACTCAGCACAGCGAA |
| *Hnf4a1* | Forward (5’ -3’) | ATGCGACTCTCTAAAACCCTTG |
|  | Reverse (5’ -3’) | ACCTTCAGATGGGGACGTGT |
| *Hprt* | Forward (5’ -3’) | CTCCTCAGACCGCTTTTTGC |
|  | Reverse (5’ -3’) | TCATCGCTAATCACGACGCT |
| *Lgr5* | Forward (5’ -3’) | AGCCTATGGACTCAATGTGAAGA |
|  | Reverse (5’ -3’) | AAATCAGCCCTAGGTCAAGATGATA |
| *Lyz1* | Forward (5’ -3’) | GAGACCGAAGCACCGACTATG |
|  | Reverse (5’ -3’) | CGGTTTTGACATTGTGTTCGC |
| *Lyz2* | Forward (5’ -3’) | ATGGAATGGCTGGCTACTATGG |
|  | Reverse (5’ -3’) | ACCAGTATCGGCTATTGATCTGA |
| *Sox9* | Forward (5’ -3’) | CGGAACAGACTCACATCTCTCC |
|  | Reverse (5’ -3’) | GCTTGCACGTCGGTTTTGG |
